# The Effect of Oxidant and the Non-Oxidant Alteration of Cellular Thiol Concentration on the Formation of Protein Mixed-Disulfides in HEK 293 Cells

**DOI:** 10.1371/journal.pone.0004015

**Published:** 2008-12-24

**Authors:** Jasen Lee Gilge, Michael Fisher, Yuh-Cherng Chai

**Affiliations:** Department of Chemistry, John Carroll University, University Height, Ohio, United States of America; Baylor College of Medicine, United States of America

## Abstract

Cellular molecules possess various mechanisms in responding to oxidant stress. In terms of protein responses, protein S-glutathionylation is a unique post-translational modification of protein reactive cysteines forming disulfides with glutathione molecules. This modification has been proposed to play roles in antioxidant, regulatory and signaling in cells under oxidant stress. Recently, the increased level of protein S-glutathionylation has been linked with the development of diseases. In this report, specific S-glutathionylated proteins were demonstrated in human embryonic kidney 293 cells treated with two different oxidative reagents: diamide and hydrogen peroxide. Diamide is a chemical oxidizing agent whereas hydrogen peroxide is a physiological oxidant. Under the experimental conditions, these two oxidants decreased glutathione concentration without toxicity. S-glutathionylated proteins were detected by immunoblotting and glutathione concentrations were determined by high performance liquid chromatography. We further show the effect of alteration of the cellular thiol pool on the amount of protein S-glutathionylation in oxidant-treated cells. Cellular thiol concentrations were altered either by a specific way using buthionine sulfoximine, a specific inhibitor of glutathione biosynthesis or by a non-specific way, incubating cells in cystine-methionine deficient media. Cells only treated with either buthionine sulfoximine or cystine-methionine deficient media did not induce protein S-glutathionylation, even though both conditions decreased 65% of cellular glutathione. Moreover, the amount of protein S-glutathionylation under both conditions in the presence of oxidants was not altered when compared to the amount observed in regular media with oxidants present. Protein S-glutathionylation is a dynamic reaction which depends on the rate of adding and removing glutathione. Phenylarsine oxide, which specifically forms a covalent adduct with vicinal thiols, was used to determine the possible role of vicinal thiols in the amount of glutathionylation. Our data shows phenylarsine oxide did not change glutathione concentrations, but it did enhance the amount of glutathionylation in oxidant-treated cells.

## Introduction

The generation of reactive oxygen species (ROS) is part of physiologically metabolic processes in cells. For example, this process can occur either in mitochondria during the electron transport chain or in NADPH oxidase of neutrophils. The redox state of cells is determined by the balance of generation of ROS and the capacity of antioxidant systems. Oxidant stress has been defined as an imbalanced redox state and favors ROS generation [1]. Oxidant stress plays a major role in many cellular responses. To understand the different mechanisms of ROS in cells, numerous studies have focused on how cellular components, lipids, proteins and nucleic acids respond to oxidant stress. ROS have been shown to trigger apoptosis, to function as signal molecules and to relate to the development of diseases [2].

In general, there are two cellular pools of thiol molecules that possess antioxidant functions. One thiol pool is composed of low molecular weight (non-protein) molecules, ascorbic acid, tocopherol and glutathione. Glutathione is the representative molecule of the non-protein antioxidant molecules because of its abundance in cells [3]. This molecule exists in two chemical forms in cells, reduced (GSH) and oxidized (GSSG) and the ratio of these two forms usually determines the redox state of cells. The metabolism of glutathione has been studied extensively in many research fields to explore the potential role of oxidant stress in different experimental conditions. A second thiol pool is composed of a long list of protein antioxidants. In addition to classic enzymes such as catalase, superoxide dismutase and glutathione peroxidase, several enzymes, such as peroxiredoxin family, have been added to that list in recent years [4]. The function and mechanism of each class of enzyme have been known and characterized. However, the relationship between these two pools in cells under oxidant stress has only been revealed recently.

Oxidative effect on proteins has received considerable attention especially cysteine residues as they are sensitive to oxidative modifications [6]. Cysteine modification can be either reversible or irreversible. Reversible modification includes disulfide formation between proteins or proteins forming mixed-disulfides with low molecular weight thiols. The latter modification plays a major role in regulating enzyme activities [7] and protein structures [8]. S-glutathionylation, formerly known as S-thiolation, is the formation of protein mixed-disulfides with glutathione. Irreversible modification occurs when protein cysteine residues are oxidized to sulfinic and cysteic acids [9] and this modification usually leads to protein degradation. Protein S-glutathionylation serves a unique role by connecting the pools of non-protein and protein thiols in cells under oxidant stress.

It is known that protein post-translational modifications play a significant role in many biochemical functions. The best example of various modifications is protein phosphorylation and dephosphorylation. Although protein S-glutathionylation is a new addition to the list of modifications, a large body of data has shown the importance of glutathionylation. Initially, many in vitro studies have shown glutathionylation is a switch to turn on/off enzymes' activities [10]. Recently, glutathionylation has been proposed as a protective mechanism in vivo to prevent enzymes from irreversible damage by oxidant stress [11]. Moreover, protein S-glutathionylation also has been shown to be involved in signal transduction [12] and the progression of disease [13]. A number of S-glutathionylated proteins have been identified in vivo [14], i.e. actin. This line of research enlarges the understanding of novel functions associated with these glutathionylated proteins. The details of the role of glutathionylation in many cellular aspects can be found in recent reviews [15], [16]. The amount of glutathionylation on proteins is a dynamic process under oxidative stress. The mechanism for the addition of glutathione to protein reactive cysteine residues is still somewhat ambiguous, but the mechanism of deglutathionylation (removing glutathione from glutathionylated proteins) is well characterized [17]. For example, glutaredoxin, a dethiolase, is known to specifically catalyze the reversed reaction of glutathionylated proteins [18].

In this report, our data shows specific S-glutathionylated proteins in HEK 293 cells using different oxidants. Since S-glutathionylation is the linkage of two cellular thiol pools, we also show the amount of protein S-glutathionylation in response to the alteration of cellular thiol pools by two different means. Futhermore, our results suggest the role of dethiolase in contributing to the amount of glutathionylation. Finally, the possible rational of the specific protein S-glutathionylation is discussed.

## Results and Discussion

### The pattern of protein S-glutathionylation in HEK 293

**Figure 1:**
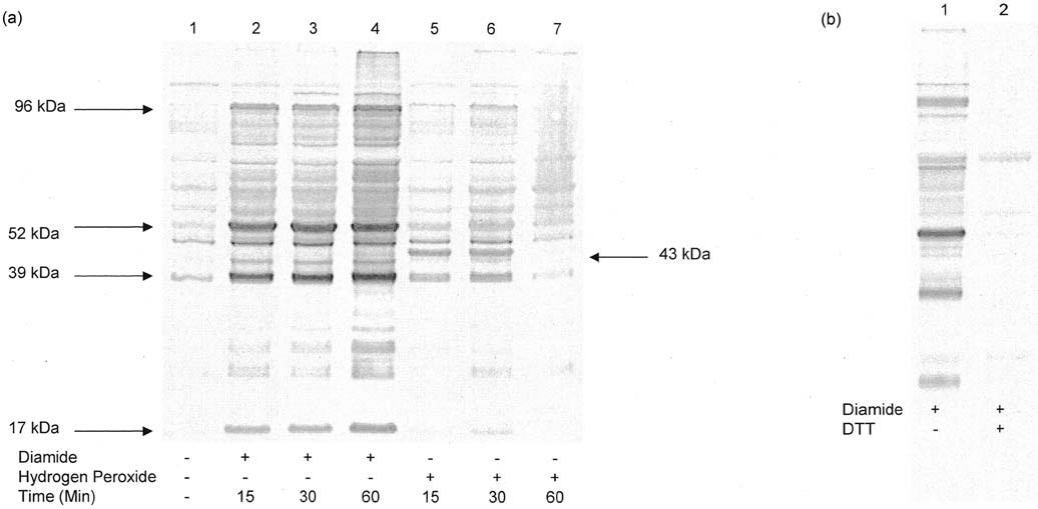
Protein S-glutathionylation in HEK 293 cells. (A) The pattern of overall S-glutathionylated proteins is shown in a time course experiment with 0.5 mM diamide or 0.5 mM hydrogen peroxide-treated HEK 293 cells. Equal amount of cell lysates were loaded and separated by a 12% SDS gel under non-reducing condition. S-glutathionylated proteins were detected by Western blot using anti-glutathione monoclonal antibody. Some predominately modified proteins are indicated by arrows. (B) The same amount of lysates from 1(A) were separated in a SDS-gel under reducing condition (50 mM DTT) and the membrane was blotted and stained the same way as in 1(A). The loss of signals indicated the anti-glutathione monoclonal antibody was selectively detecting glutathione moiety on proteins.

Although protein S-glutathionylation has been studied by various means [23], the traditional method, SDS-PAGE with autoradiograph or Western bolt, is still one of the most widely used tools to detect the modification. The validity of anti-glutathione monoclonal antibody has been shown in several studies [24], [25], including our own [26]. In Figure 1 A, we show the pattern of S-glutathionylation in HEK 293 cells treated with two oxidants, diamide and hydrogen peroxide. Cell extracts were made from intact cells with lysis buffer containing freshly prepared N-ethylmaleimide, a specific thiol-akylating agent, to block any artifacts during sample preparations. There were basal modifications in untreated cells (Figure 1A, lane 1) and the amount of modification was enhanced by the addition of two oxidants to cells. Diamide, a well-known chemical oxidizing agent [27], was chosen as a positive control to induce all possible proteins that undergo S-glutathionylation in HEK 293. Also, we show the pattern of S-glutathionylation by a physiological oxidizing agent, hydrogen peroxide (Figure 1A, lane 5–7). It is not surprising that hydrogen peroxide induces fewer modifications than diamide. Diamide shows an increase of overall modifications with time, whereas hydrogen peroxide shows a decrease of overall modifications with time. With each oxidant, proteins responded in different degrees of modifications, i.e. some proteins were more sensitive than others. Some predominately modified proteins are indicated by arrows. The glutathione metabolism in the presence of these two oxidants is shown in Table 1. There was a total glutathione decrease by both oxidants and there were still more reduced GSH than GSSG at each time point. Interestingly, a unique S-glutathionylated protein band (43 kDa) was observed only induced by hydrogen peroxide. The signals of protein bands were reduction sensitive, in the presence of DTT, indicating that this monoclonal antibody selectively detected protein mixed-disulfided with glutathione (Figure 1B).

**Table 1:** The Effect of Diamide (0.5 mM) and Hydrogen Peroxide (0.5 mM) on Glutathione Concentration.

Treatment	GSH	GSSG	Total GSH*
	(n^.^moles/mg protein)	(n^.^moles/mg protein)	(n^.^moles/mg protein)
Untreated	30.19	0.40	30.99
Diamide 15 min	20.25	0.91	22.07
Diamide 30 min	19.87	0.65	21.17
Diamide 60 min	30.42	0.85	32.12
H_2_O_2_ 15 min	30.84	0.74	32.32
H_2_O_2_ 30 min	27.17	0.79	28.75
H_2_O_2_ 60 min	25.73	1.08	27.89

* Total GSH is calculated by [GSH]+2[GSSG].

The overall pattern of S-glutathionylation suggests different degrees of sensitivities of proteins in response to oxidative stress. The predominately S-glutathionylated proteins may be due to the number of reactive cysteine residues (these cysteine residues with lower pKa values) associated with protein, or simply, the reflection of the abundance of a protein level. Based on the presented data, our results suggest that different oxidative mechanisms stimulate different sets of proteins responding to stress. In our studies, there were significant amounts of S-glutathionylation, even in the presence of more reduced GSH than GSSG. Our data supports a previously proposed mechanism [28] where glutathione disulfide is not a prerequisite factor for the formation of S-glutathionylation in cells.

In theory, diamide should induce all possible proteins that undergo S-glutathionylation. However, a significantly modified protein (43 kDa) is only induced by hydrogen peroxide. These data suggest the 43 kDa protein is only sensitive to a free- radical related mechanism because another oxidant, *t*-Butyl hydroperoxide also induced 43 kDa S-glutathionylation in HEK 293 cells (data not shown). To our knowledge, this is the first evidence that shows specific protein S-glutathionylation by different oxidative mechanisms in cells. It is necessary to determine the identity of this 43 kDa protein to understand its function and propose a mechanism for the specific S-glutathionylation by hydrogen peroxide. We speculate that this 43 kDa protein could be actin since actin has been shown as a target protein for S-glutathionylation [24].

### The effect of cellular thiol concentration on protein S-glutathionylation

**Figure 2:**
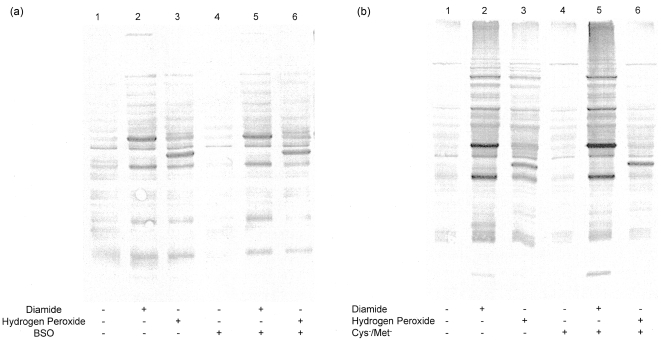
The relationship between the amount of glutathionylation and the cellular thiol concentration. (A) Protein S-glutathionylation in BSO-treated cells. HEK 293 cells were incubated with 100 µM BSO overnight before treating with 0.5 mM diamide or 0.5 mM hydrogen peroxide. The cellular glutathione concentration was decreased 58% (Table 2) compared to untreated cells. Cell lysates were prepared and modified proteins were detected as described under Methods. There was no enhanced protein S-glutathionylation in BSO-treated alone cells, i.e. no addition of oxidants. (B) Protein S-glutathionylation in cells incubated with cystine-methionine deficient media. HEK 293 cells were placed in cystine-methionine deficient media overnight before treating with 0.25 mM diamide or 0.5 mM hydrogen peroxide. The cellular glutathione concentration was decreased 64% (Table 2) compared to untreated cells. Cell lysates were prepared and modified proteins were detected as described under Methods. Again, there was no enhanced protein S-glutathionylation in cells alone placed in cystine-methionine deficient media.

Protein S-glutathionylation serves as the linkage between the pools of protein and non-protein thiols. We took two approaches to determine whether the amount of S-glutathionylation changes if the pools of cellular thiols are altered. Since glutathione is the most abundant non-protein antioxidant molecule in cells, we studied the effect of cellular glutathione concentration on protein S-glutathionylation. Buthionine sulfoximine (BSO) irreversibly inhibits gamma-glutamylcysteine synthetase, a required enzyme for GSH biosynthesis, thereby depleting cells of glutathione. Cells were incubated with BSO (100 µM) overnight before treating with oxidants. In Table 2, the BSO-treated cells showed 58% less cellular glutathione relative to the untreated cells. The effect of BSO on S-glutathionylation is shown in Figure 2A, lane 4–6. First, we expected the amount of protein glutathionylation would decrease in BSO and oxidant-treated cells due to the loss of glutathione from BSO pretreatment. On the contrary, our data shows the overall modifications in these cells were not altered, even though the glutathione concentration was decreased 58%. Secondly, we expected to see the enhanced protein S-glutathionylation in BSO-treated alone cells (i.e., no addition of oxidants) (Figure 2A, lane 4) since decreasing glutathione concentration reflects the presence of amplified oxidant stress. Interestingly, there were no enhanced modifications in these cells.

**Table 2:** The Effect of BSO and Cystine-Methionine Depleted Medium on Glutathione Concentration.

Treatment	GSH	GSSG	Total GSH
	(n^.^moles/mg protein)	(n^.^moles/mg protein)	(n^.^moles/mg protein)
Untreated	30.99	0.30	31.58
100 µM BSO	12.87	0.20	13.26
Cystine-Methionine Depleted Medium	11.32	0.13	11.59

Another non-specific approach was used to examine the effect of cellular thiol concentrations on protein S-glutathionylation. Cells were incubated in cystine-methionine deficient media overnight before treating with oxidants. This is a non-specific method to deplete thiol concentration versus the specific method using BSO. The data in Table 2 show the use of cystine-methionine deficient media decreased cellular glutathione concentration by 64% compared to regular media. However, the amount of S-glutathionylation on proteins did not correlate with the loss of glutathione (Figure 2B, lane 4–6) in cystine-methionine deficient and oxidant-treated cells. Again, there was no augmentation of protein S-glutathionylation in cystine-methionine deficient media alone cells (Figure 2B, lane 4).

Our data also shows two different mechanisms to deplete cellular glutathione: oxidative (diamide and hydrogen peroxide) and non-oxidative (BSO and cystine-methionine deficient medium). Although the non-oxidative mechanism depletes approximately 60% of cellular glutathione, only protein S-glutathionylation can be detected in an oxidative mechanism. A possible explanation for the amount of protein S-glutathionylation in BSO and cystine-methionine deficient media-treated cells is that there are still enough glutathione for glutathionylation under these conditions. Either BSO or a cystine-methionine deficient medium depletes approximately 60% cellular glutathione. Upon adding oxidants, the remaining 40% of glutathione was able to form the same amount of S-glutathionylation found in non-depleted cells to prevent proteins from irreversible damage. Furthermore, the 40% glutathione observed may also be enough to maintain a physiological redox environment in cells, i.e. most proteins are still in reduced state. This could be the reason why protein S-glutathionylation was not enhanced in BSO and cystine-methionine deficient medium alone cells.

### The possible role of vicinal dithiols in protein S-glutathionylation

**Figure 3:**
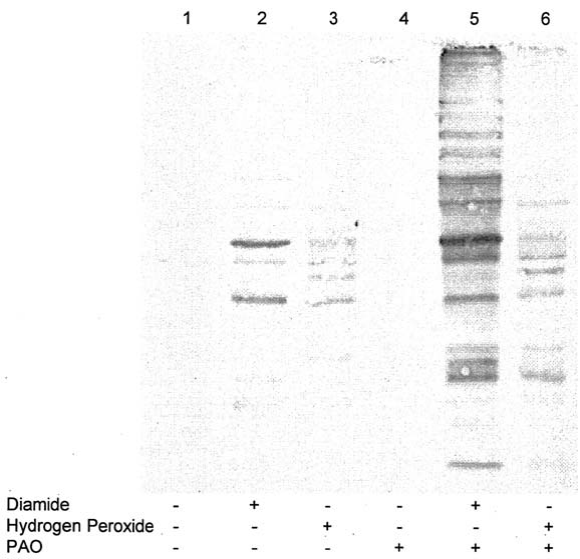
The relationship between the amount of S-glutathionylation and the vicinal dithiols-containing molecules. HEK 293 cells were pretreated with 1 µM PAO for 30 minutes before treating with 0.25 mM diamide or 0.5 mM hydrogen peroxide. The preparation of PAO was described under Methods, and the effect of PAO on cellular glutathione was shown in Table 3. Cell lysates were prepared, and modified proteins were detected as described under Methods.

Protein S-glutathionylation is known to be an equilibrium process that depends on the rate of adding glutathione and the rate of removing glutathione, i.e. dethiolation. Since the mechanism for the addition of glutathione is not well understood, experiments were performed to study the possible role of removing glutathione on the amount of S-glutathionylation. Dethiolation has been shown to be an enzyme-dependent reaction; the enzyme contains vicinal dithiols. Phenylarsine oxide (PAO) is a specific vicinal dithiol-binding agent and hardly binds to monothiols. Cells were pretreated with a sub-toxic concentration of PAO before oxidant treatment. The data in Table 3 show that PAO (1 µM) did not alter the cellular glutathione concentration as expected (since GSH is a monothiol molecule). However, PAO did significantly enhance the amount of glutathionylation in diamide and hydrogen peroxide-treated cells (figure 3A, lane 5–6). The lower concentration of diamide was used in this experiment in order to detect the enhancement of modification by PAO. Despite the different oxidative mechanisms of diamide and hydrogen peroxide, our results suggest that vicinal-dithiol containing molecules may play a role in the regulation of protein S-glutathionylation in both oxidant-treated cells.

**Table 3:** The Effect of PAO (1.0 µM) on Glutathione Concentration.

Treatment	GSH	GSSG	Total GSH
	(n^.^moles/mg protein)	(n^.^moles/mg protein)	(n^.^moles/mg protein)
Untreated	31.98	0.31	32.59
0.25 mM Diamide 30 min	13.05	3.02	19.08
0.5 mM H_2_O_2_ 30 min	30.51	1.65	33.81
1.0 µM PAO	33.66	0.37	34.40
PAO, 0.25 mM Diamide 30 min	7.62	3.77	15.16
PAO, 0.5 mM H_2_O_2_ 30 min	21.70	3.71	29.13

Although our data show the specific effect of PAO under the experimental condition, the depletion of glutathione by oxidants was more dramatic in PAO-pretreated cells than non-pretreated cells. This observation suggests some vicinal-dithiol containing molecules play an antioxidant role in HEK 293 cells. The potential target for the PAO effect in our condition could be glutaredoxin since it is a known dethiolase. Glutaredoxin contains vicinal dithiols in the catalytic site and its function is usually characterized in vitro using purified S-glutathionylated proteins. To our knowledge, this is the first data to show increased S-glutathionylation by possibly inhibiting the activity of dethiolase (glutaredoxin) in vivo. Our in vivo data also support the perception that protein S-glutathionylation is a dynamic reaction. In future experiments, we will attempt to assay the activity of glutaredoxin in PAO-treated cells to reveal the role of glutaredoxin in protein S-glutathionylation in vivo.

## Materials and Methods

Dithiothreitol (DTT), *N*-ethylmaleimide (NEM), DL-buthionine sulfoximine (BSO), Dulbecco's modified eagle's medium (DMEM), cystine-methionine deficient DMEM, glutathione disulfide (GSSG), reduced glutathione (GSH), iodoacetic acid (IAA), 1-flouro-2,4-dinitrobenzene (FDNB), diamide, fetal bovine serum (FBS), and phenylarsine oxide (PAO) were all purchased from Sigma-Aldrich (ST. Louis, MO). Goat anti-mouse antibody conjugated with alkaline phosphatase and its substrate were purchased from BioRad (Hercules, CA). Anti-glutathione monoclonal antibody was purchased from Virogen (Watertown, MA). Hydrogen peroxide was purchased from Calbiochem, a brand of EDM Bioscience, Inc (La Jolla, CA). Waters Spherisorb NH_2_ (5 µm 250 mm×4.6 mm) high performance liquid chromatography (HPLC) ion exchange column was purchased from Supelco Inc. (Bellefonte, PA). Human embryonic kidney cells (HEK 293) were kindly provided by Dr. Anthony J. Koleske (Yale University).

### Preparation and identification of S-glutathionylated proteins in vivo

HEK 293 cells were seeded into 60 mm culture plates and grown until 70% confluent in Dulbecco's modified eagle's medium (DMEM) containing 8% fetal bovine serum (FBS) at 37°C supplemented with 5% carbon dioxide. The medium was changed to 0.5% FBS before the treatment of oxidants so the oxidant's effect would not be quenched by FBS.

S-glutathionylation was induced using two oxidants: diamide and hydrogen peroxide. After the oxidant treatment, the cells were harvested in a lysis buffer (10 mM tris-HCl, 150 mM NaCl, 1 mM EGTA, 1% Nonidet P40, 1 mM EDTA, pH 7.4) containing freshly prepared 50 mM *N*-ethylmaleimide (NEM). NEM irreversibly alkylates thiol groups to prevent further S-glutathionylation during sample processing. Soluble fractions of cell lysate were separated by centrifugation at 4°C and protein concentration was determined via Bradford Assay [19]. Equal amounts of proteins were electrophoretically separated by a 12% SDS gel in the absence of reducing agent proceeded by Western Blotting. S-Glutathionylated proteins were detected by Western Blot using an anti-glutathione monoclonal antibody and the color reaction was performed according to the instruction manual from BioRad. All SDS gels were run with the same pre-stained molecular weight standards from BioRad. The estimation of molecular weight of S-glutathionylated proteins was normalized using the molecular weight of standard proteins. The specificity of this monoclonal antibody was confirmed by running samples under reducing conditions. Equal amounts of cell lysates were incubated with 50 mM DTT before running SDS-gel and Western blotting. The loss of signals in the presence of DTT indicated that the glutathione molecules formed disulfides with proteins.

Cells were pretreated with BSO (100 µM) for 16 hours to alter cellular thiol pools prior to medium change. BSO specifically inhibits glutathione synthesis by inhibiting gamma-glutamylcysteine synthetase [20]. Similarly, cells were incubated with cystine-methionine deficient DMEM containing 8% FBS for 16 hours prior to changing the medium to cystine-methionine deficient DMEM containing 0.5% FBS. PAO reacts with vicinal dithiol containing molecules [21]. Cells were pretreated with PAO (1 µM) for 30 minutes before oxidant treatment. PAO was dissolved in dimethyl sulfoxide (DMSO) and the final concentration of DMSO in media was 0.1%. S-Glutathionylated proteins in these conditions were prepared and analyzed as described above.

### HPLC determination of glutathione and glutathione disulfide concentration

After oxidant treatment, the cells were harvested in 5% perchloric acid for 20 min on ice. Acid soluble and insoluble fractions were separated by centrifugation. Acid soluble fractions were used to determine GSH and GSSG concentration and acid insoluble fractions were used to determine the protein concentration. Glutathione samples were prepared and determined by a previously described method [22]. Briefly, acid-soluble fractions were reacted with iodoacetic acid at neutralized pH to block free sulfhydryal groups. Finally, 1-flouro-2,4-dinitrobenzene (FDNB) was added to the mixture for the detection at 365 nm. Both reduced and oxidized glutathione forms were separated by HPLC according to a published method [22]. Synthetic GSH and GSSG with known concentrations were prepared under the same method for identification and quantification of cellular GSH and GSSG. The cellular GSH and GSSG concentrations were calculated from a standard curve and normalized by the amount of protein in each sample.
